# Bibliographical

**Published:** 1866-01

**Authors:** 


### BIBLIOGRAPHICAL.

Instructions in the Manipulation of Hard Rubber, or Vulcanite, for Dental Purposes, by Prof. E. Wildman, of Penn. College of Dental Surgery.—This very excellent treatise upon this subject, has appeared at a time when it is very much needed.

The introduction of hard rubber as a base for artificial teeth, has become very general in the profession, and yet previous to this issue, there has been no full and concise instructions in regard to working it. Perhaps no one in this country is so well qualified to prepare a work of this kind as Prof. Wildman. Certainly none stand higher than he in that department of practice, to which he has given such close attention. He has completed a larger range of experiments upon the vulcanization of gums, than any other one in this country. This work is written in a clear, understandable manner; it is one that he who is seeking instruction in this department will easily comprehend, and prize highly; indeed we would recommend it to all who work rubber, or desire to do so, for the neat, systematic, and efficient method described in this work, very few in the profession work up to.

The work is published in a very neat form, by S. S. White, and may be obtained of him or any of his agents.

